# Mitochondria as a target for neuroprotection: role of methylene blue and photobiomodulation

**DOI:** 10.1186/s40035-020-00197-z

**Published:** 2020-06-01

**Authors:** Luodan Yang, Hannah Youngblood, Chongyun Wu, Quanguang Zhang

**Affiliations:** 1 Department of Neuroscience and Regenerative Medicine, Medical College of Georgia, Augusta University, 1120 15th Street, Augusta, GA 30912 USA; 2 Department of Cellular Biology and Anatomy, Medical College of Georgia, Augusta University, 1120 15th Street, Augusta, GA 30912 USA

**Keywords:** Mitochondrial dysfunction, Neuroprotection, Methylene blue, Photobiomodulation

## Abstract

Mitochondrial dysfunction plays a central role in the formation of neuroinflammation and oxidative stress, which are important factors contributing to the development of brain disease. Ample evidence suggests mitochondria are a promising target for neuroprotection. Recently, methods targeting mitochondria have been considered as potential approaches for treatment of brain disease through the inhibition of inflammation and oxidative injury. This review will discuss two widely studied approaches for the improvement of brain mitochondrial respiration, methylene blue (MB) and photobiomodulation (PBM). MB is a widely studied drug with potential beneficial effects in animal models of brain disease, as well as limited human studies. Similarly, PBM is a non-invasive treatment that promotes energy production and reduces both oxidative stress and inflammation, and has garnered increasing attention in recent years. MB and PBM have similar beneficial effects on mitochondrial function, oxidative damage, inflammation, and subsequent behavioral symptoms. However, the mechanisms underlying the energy enhancing, antioxidant, and anti-inflammatory effects of MB and PBM differ. This review will focus on mitochondrial dysfunction in several different brain diseases and the pathological improvements following MB and PBM treatment.

## Background

There are several common causal factors for brain disorders, including oxidative stress, inflammation, transcriptional alterations, and excitotoxicity. Mitochondrial dysfunction plays a central role in the induction of these factors which lead to neurological disorders [1]. As a result, mitochondrial dysfunction has been the subject of several recent investigations studying the pathophysiology of neurological disorders [2–5]. In neurological disorders such as Alzheimer’s disease (AD), traumatic brain injury (TBI), depression, stroke, and Parkinson’s disease (PD), mitochondria contribute to the pathophysiology through decreased energy production and excessive production of reactive oxygen species (ROS) [6, 7]. Research on mitochondrial dysfunction in brain diseases indicate that restoration of mitochondrial function is a potential method for treatment of neurodegeneration and other brain disorders [8–12]. Numerous studies indicate an improvement of brain mitochondrial function following treatment for neurological illness [13–15]. PBM and MB, the most widely studied approaches targeting mitochondrial respiration, were recognized as promising prospective treatments for brain disorders [16–20]. However, the mechanisms underlying the energy enhancing, antioxidant, and anti-inflammatory effects of MB and PBM differ. Because PBM and MB exert beneficial effects through distinct mechanisms, combining the use of these two therapies is expected to improve therapeutic outcomes in a synergistic manner.

## Main text

### Mitochondrial structure and function

**Fig. 1:**
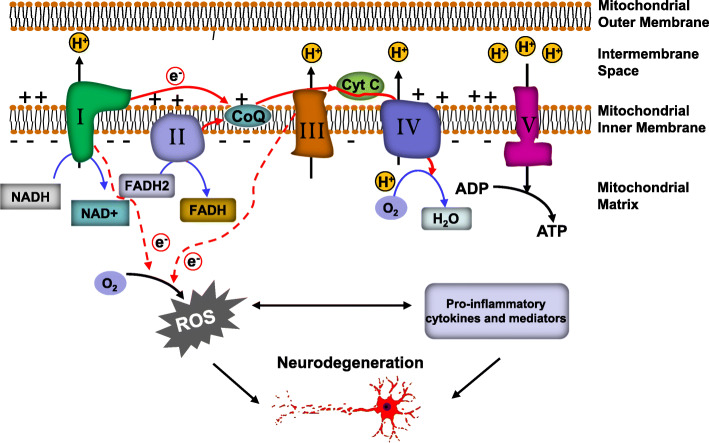
Diagram of electron leakage in brain disease. Electrons in the mitochondrial ETC are transferred along a series of four protein complexes (Complexes I-IV) with the aid of electron transporters NADH, FADH2, ubiquinone (Co-enzyme Q10, CoQ), and cytochrome c (Cyt c). As a result of this electron transfer, protons are pumped by Complexes I, III, and IV from the mitochondrial matrix into the intermembrane space, thereby generating an electrochemical gradient across the inner mitochondrial membrane. This gradient is used to propel ATP synthase (Complex V) to produce ATP. Although this process is highly efficient, electrons can escape from Complex I and Complex III and be transferred to O_2_, which is reduced to the radical O2•−. This ROS production is exacerbated under pathological conditions such as brain disease and activates inflammatory processes, thereby establishing a cycle of ROS production, inflammation, and neuronal damage

Mitochondria are organelles found in eukayotic cells and are characterized by a double membrane structure [21]. In eukaryotic cells, the mitochondria generate the majority of adenosine triphosphate (ATP), which provides energy for cellular activities. The mitochondrial electron transport chain (ETC) is centrally involved in this ATP production. Five multi-subunit complexes in the mitochondrial inner membrane form the respiratory chain for oxidative phosphorylation [22]. As shown in Fig. 1, Complex I (NADH–ubiquinone oxidoreductase) and Complex II (succinate–ubiquinone oxidoreductase) work as the major entrance points for electrons into the respiratory chain. NADH and FADH2 transfer their electrons to Complex I and Complex II, respectively. These electrons flow between the complexes down an electrochemical gradient, shuttled by Complexes III and IV and by two mobile electron carriers, ubiquinone (Co-enzyme Q10, CoQ) and cytochrome c (Cyt c) [23]. In the process of transfer, a proton motive force is established by pumping the protons through the inner mitochondrial membrane to the intermembrane space. Complex V (ATP synthase) depends on this process to generate ATP by phosphorylating ADP [24, 25].

A large body of evidence demonstrates that several changes induced by mitochondria, including oxidative stress, Ca^2+^ imbalance, dysfunctional electron transport, impaired mitochondrial trafficking, altered mitochondrial dynamics, and defective mitophagy, are involved in various brain diseases (Table 1) [32, 62, 79, 105–108]. Among these mitochondrial changes, oxidative stress and inflammation are the most directly related factors affecting the survival of neurons [109].

**Table 1:** Summary of mitochondria-related changes in brain disease

Condition of interest	Observed mitochondria-related changes in brain disease
**Alzheimer ‘s disease**	• Increased ROS production [26, 27]
	• Impaired balance of mitochondrial fission and fusion [8, 28–31]
	• Aberrant mitochondrial enzymes [32–35]
	• Increased mtDNA mutation [36]
	• Abnormal function of mitochondrial import channels [37]
	• Inflammation [38, 39]
	• Accumulation of APP/Aβ in mitochondrial import channels [37]
	• Mitochondrial dysfunction-induced apoptosis [6, 40, 41]
	• Impaired Na^+^/Ca^2+^ exchanger (mitochondrial Ca^2+^ overload) [42–44]
	• Impaired mitochondrial trafficking [45–47]
	• Mitophagy defects [48, 49]
**Traumatic Brain Injury**	• Decreased mitochondrial membrane potential [50]
	• Mitochondrial Ca^2+^ overload [50, 51]
	• Reduced oxidase complex activity [52]
	• Imbalance of mitochondrial fusion and fission induced mitochondrial respiration dysfunction, increased ROS production, and release of apoptosis- related factors [53–57]
	• Impaired mitopahgy [58]
**Stroke**	• Failure of membrane ion pump, cellular potassium efflux, sodium influx, and the depolarization of the membrane [59–61]
	• The dysregulation of mitochondrial Ca^2+^ homeostasis [62–64]
	• Cytochrome c release induced apoptosis [65, 66]
	• Excessive mitochondrial superoxide production [67–69]
	• Mitochondrial dynamics defects [70–74]
	• Abnormal mitophagy [75–77]
**Depression**	• Inhibition of mitochondrial OXPHOS activity [78]
	• Decreased content of mitochondrial enzymes [79, 80]
	• Inhibition of complexes in the mitochondrial respiratory chain and the activity of Na+, K + -ATPase [81–85]
	• Increased mtDNA mutation [78, 86, 87]
	• Impaired mitochondrial ETC [78, 88, 89]
**Parkinson’s disease**	• Mitochondrial respiration defects [90, 91]
	• Genetic mutation induced mitochondrial dysfunction [92–95]
	• Excessive ROS production [90, 96, 97]
	• Mitochondrial dynamics defects [98–100]
	• Mitochondrial Ca^2+^ overload in DA neurons [101, 102]
	• Inappropriate trafficking of damaged mitochondria [103]
	• Compromised mitophagy [104]

### Mitochondrial dysfunction

Mitochondria are the primary source of ROS, as they use oxygen for energy production. As mentioned previously, the mitochondrial ETC is composed of five protein complexes, which are located in the inner mitochondrial membrane. The coenzymes NADH and FADH2 are responsible for electron transport along the ETC. This process is coupled with the transport of protons across the mitochondrial inner membrane, thereby forming an electrochemical proton gradient, which is used to drive ATP synthesis at Complex V. Although the process is relatively efficient, about 0.4 to 4% oxygen is incompletely reduced and produces ROS [10]. Under normal metabolic conditions, the main site of ROS generation is Complex III [110].

Certain levels of ROS are necessary for normal cellular function and healthy physiological processes, including response to anoxia, cellular signaling pathways, and the induction of a mitogenic response [111, 112]. As reported, ROS signaling can lead to a cascade of cell-to-cell communication, which allows a signal to propagate over long distances through different tissues [113]. Furthermore, the important role of physiological levels of ROS is exemplified in the immune system of granulomatous patients. Due to defects in the NADPH oxidase system, these patients cannot produce enough ROS to protect themselves from the persistent infections seen in many cases [112]. As mentioned above, ROS produced by mitochondria work as signaling molecules in response to stress, whereby transcriptional changes are initiated in the nucleus [114]. These transcriptional changes induce the increased expression of proteins contributing to improved systemic defense.

Although certain levels of ROS are beneficial, oxidative stress, characterized by excessive ROS production, has been recognized as a major contributing factor to the pathophysiology of several neurodegenerative diseases [10]. Mitohormesis describes the phenomenon in which low levels of ROS can be protective and beneficial while high levels of ROS can be deleterious [111, 112].

The production of ROS under various disease states overwhelms the maximum ability of defense mechanisms, wherein the excessive ROS production induces oxidative damage to lipids, proteins, and nucleic acids in the cell [115, 116]. Because the integrity of the inner mitochondrial membrane is essential to the creation of an electrochemical gradient and ETC function, oxidative damage to the lipids and proteins that comprise the membrane results in further ETC dysfunction [117]. Furthermore, the impaired ETC enhances the overproduction of ROS, thus establishing an increasingly destructive cycle of oxidative stress and respiratory chain defects [118].

In addition to its effects on the ETC, high levels of ROS can also have deleterious effects on other aspects of mitochondrial function. Ca^2+^ plays a crucial role in the activation of mitochondrial enzymes, including pyruvate dehydrogenase, NAD-linked isocitrate dehydrogenase, and 2-oxoglutarte dehydrogenase [119]. Mitochondria can uptake Ca^2+^ into the mitochondrial matrix via the mitochondrial calcium uniporter complex (MCU) [120]. This process is driven by ATP hydrolysis and the electrochemical gradient produced by the ETC and regulates mitochondrial metabolism, cytoplasmic Ca^2+^ signaling, and cell death [121]. Under normal conditions, the uptake of calcium enhances mitochondrial respiratory function and tunes mitochondrial function to synaptic activity [122, 123]. However, excessive ROS generation can disturb Ca^2+^ homeostasis and induce Ca^2+^ overload. Elevation of Ca^2+^ levels can cause a change in mitochondrial potential and induce further production of ROS [124]. During this process, the mitochondria may undergo a collapse of membrane potential, increased mitochondrial permeability, and a rupture of the outer mitochondrial membrane [125]. The increased membrane permeability will finally result in cytochrome c release, thereby initiating cellular apoptosis [109, 121, 126].

Overproduction of ROS can also induce mitophagy by causing mitochondrial damage [127]. Mitophagy is the selective degeneration of mitochondria by autophagy in response to mitochondrial stress or damage [128]. During this process, the sustained depolarization of the inner membrane induces the accumulation of PTEN-induced kinase 1 (PINK1) at the outer mitochondrial membrane [129]. The accumulation of PINK1 causes the phosphorylation of mitofusin 2 (Mfn2), the recruitment of Parkin to the outer mitochondrial membrane, the subsequent formation of phosphor-ubiquitin chains on mitochondrial outer membrane proteins, and the recruitment of autophagy receptors (e.g. OPTN, NDP52) [130]. These receptors bind to ubiquitin and LC3 forming an autophagosome [128]. Increasing evidence suggests that the inhibition of mitophagy results in mitochondrial dysfunction [128]. Impaired mitophagy has been demonstrated in most neurodegenerative diseases, including AD, PD, amyotrophic lateral sclerosis (ALS), and Huntington’s disease [131, 132]. Impaired mitophagy cannot efficiently remove damaged mitochondria, which in turn enhances the overproduction of ROS and exacerbates the hyperinflammation induced by excessive ROS [132, 133].

In addition to mitophagy, the balance between mitochondrial fusion and fission (i.e. mitochondrial dynamics) has been recognized as a quality control mechanism. As a result, impaired mitochondrial dynamics has been considered as one of the intrinsic causes of mitochondrial dysfunction [134]. Evidence suggests that oxidative stress is able to cause excessive mitochondrial fission, which contributes to mitochondrial dysfunction [135, 136]. Furthermore, studies have found that knock-down of fission promoting protein Drp1 is able to attenuate the generation of pro-inflammatory factors and the production of ROS, indicating an important role for mitochondrial dynamics in protection against cellular damage [137].

In addition, a large body of evidence supports a cyclic relationship between mitochondrial dysfunction and inflammation [138]. It has been well established that mitochondrial perturbation and the release of mitochondrial components into the cytosol are able to induce innate immune response activation, which leads to increased release of inflammatory mediators [139–141]. Zhou et al., reported that ROS-generating mitochondria are able to induce inflammation through NLRP3 inflammasome-dependent inflammatory pathways [142]. Furthermore, the NLRP3 inflammasome complex can work as a mitochondrial dysfunction sensor [142]. This innate immune activation may occur due to a mitochondrial dysfunction prompted release of damage-associated molecular patterns (DAMPs) [143].

Mitochondrial DNA (mtDNA), a type of mitochondrial DAMP, can elicit a strong inflammatory response as it shares similarities to bacterial DNA [144]. As mentioned previously, under normal conditions, mitophagy is responsible for the degradation of defective mitochondria [128]. However, in brain disease, overproduction of ROS can result in impaired mitophagy which allows damaged mitochondria to go unchecked [145]. These defective mitochondria may eventually have a loss of membrane integrity allowing mtDNA to escape into the cytosol [146]. As a result, mtDNA may induce the release of type I interferon and the expression of other interferon-driven genes [147, 148].

Conversely, inflammation can also impact mitochondrial function. For example, IL-1β has been shown to induce mitochondrial fragmentation and decrease mitochondrial respiration rate via the mitochondrial fission-related protein Drp1 [149]. Furthermore, mitochondria treated with tumor necrosis factor alpha (TNFα) have been shown to be smaller, more condensed, and hollow with fewer inner membrane folds (i.e. cristae). In addition, these mitochondria had a lower membrane potential, decreased intracellular ATP production, and significantly increased ROS generation [150]. Moreover, mitophagy increased in neuroblastoma cells following TNF treatment, suggesting an interplay between inflammation and mitophagy [151]. Taken together, this evidence provides strong support for a cyclic relationship between inflammation and mitochondrial dysfunction.

### Mitochondrial dysfunction in Alzheimer ‘s disease

Alzheimer’s Disease (AD) is a progressive, age-dependent neurodegenerative disorder, characterized by progressive impairment of learning and memory, formation of neurofibrillary tangles (NFT), and extracellular deposition of amyloid-β (Aβ) [152, 153]. In addition to these pathological hallmarks [153, 154], numerous studies have indicated that mitochondrial abnormalities are involved in the development of AD. For example, hypometabolism, decreased cerebral blood flow, decreased oxygen extraction, and decreased oxygen utilization have been observed in AD patients [155–157]. In addition, increased oxidative stress and ROS production have also been observed in the brains of AD patients [158]. The observed increase in ROS generation is capable of inducing DNA, proteins, and lipid damage, a typical pathological signature in AD neural tissue [26, 27].

In addition to decreased regional blood flow and oxygen utilization, an impaired balance of mitochondrial fission and fusion has also been observed in the brains of AD patients [28, 29]. The expression of mitochondrial fusion proteins, such as Opa1, Mfn1, and Mfn2, were downregulated, while the levels of the fission proteins Drp1, Fis1, Mff, and Mief were upregulated in AD, suggesting that an elevated ratio of fission over fusion proteins may have been contributing to mitochondrial and neuronal dysfunction [8, 28, 30, 31].

Furthermore, previous studies have found that aberrant mitochondrial enzymes, including Complex IV, pyruvate dehydrogenase complex, and the α-ketoglutarate dehydrogenase complex, contribute to the development of AD [32–35]. One of the mechanisms underlying aberrant mitochondrial enzyme expression is the accumulation of mutations in the mtDNA sequences that code for subunits of these enzymes. Coskun et al., has shown that mtDNA mutations are more prevalent in AD patients than in healthy subjects [36]. In addition, reduced transcription of mtDNA L-strand ND6 and a decreased mtDNA/nuclear DNA ratio in AD patients has been suggested to decrease oxidative phosphorylation, and therefore, may account for some of the mitochondrial abnormalities observed in AD [36].

Moreover, mitochondrial calcium (mCa^2+^) signaling is a key regulator in the aberrant mitochondrial enzyme-induced metabolic dysfunction associated with AD. In a mouse model of AD, deletion of the Na^+^/Ca^2+^ exchanger precedes memory deficits and significantly increased amyloidosis and tau hyperphosphorylation via metabolic dysfunction and excessive superoxide production, thereby suggesting that defective mCa^2+^ signaling contributes to AD progression [42, 43]. The Na^+^/Ca^2+^ exchanger is essential for proper nerve firing due to its removal of Ca^2+^ from nerve terminals [45].

Therefore, the number and functionality of mitochondria at axon terminals is critical. Impaired mitochondrial trafficking has been observed in AD, and this transport defect is thought to contribute to the absence of mitochondria in presynaptic nerve terminals and may result in a diminished ATP supply in these areas [46]. The decreased local ATP supply may cause attenuated ATP-dependent vesicular neurotransmitter loading and synaptic vesicle transport [46, 47].

Additionally, as mentioned previously, mitophagy is essential for maintaining normal neuronal function through the clearance of damaged mitochondria. Numerous studies have demonstrated defective mitophagy in AD and have suggested approaches for restoring mitophagy as a strategy for ameliorating AD pathology [48, 49].

More directly, there have been studies supporting an interaction between dysfunctional mitochondria and abnormal processing of amyloid and tau. In the amyloid precursor protein (APP) transgenic mice model, APP is trapped in the mitochondrial membrane and affects mitochondrial function through the accumulation of APP/Aβ in mitochondrial import channels [159]. The obstructed mitochondrial import channels impair the function of ETC enzymes and induce accumulation of toxic hydrogen peroxide species [37]. Furthermore, mitochondrial dysfunction has been shown to incite aberrant Aβ production [160]. Chemical inhibition of mitochondrial ETC has been shown to enhance proteolytic APP processing, leading to increased levels of Aβ [160]. Another study has found that an increased intracellular Aβ level in AD-derived cybrids was accompanied by increased oxidative stress and neuronal apoptosis [161].

### Mitochondrial dysfunction in traumatic brain injury

TBI is an injury often acquired through falls, sports accidents, motor vehicle crashes, violence, and other sources of physical trauma [162]. Following the physical injury, molecular changes in the cellular microenvironment, including mitochondrial damage, excessive ROS release, decreased ATP generation, neuronal loss, neuroinflammation, dysfunction of the blood-brain barrier (BBB), and edema formation, contribute to secondary injury [163, 164]. Among these, mitochondrial dysfunction plays a crucial role in TBI-induced neuronal apoptosis and necrotic cell death. For example, mitochondrial metabolism studies have provided evidence of reduced Complex IV activity persisting for up to 14 days after TBI [52]. In addition, after TBI, apoptotic proteins (Bak/Bax) can induce mitochondrial swelling through membrane pore formation. During this process, small mitochondria-derived activator caspases are released, followed by apoptosome formation and neuronal apoptosis [163].

TBI causes both a continual release of excitatory neurotransmitters and an impairment, or even a reversal, of neurotransmitter uptake mechanisms, resulting in excessive levels of neurotransmitters in the synaptic cleft and subsequent excitotoxicity [165]. Voltage-dependent Ca^2+^ channels are one of the main mechanisms mediating excitotoxicity [165]. To buffer the influx of Ca^2+^ through these channels, mitochondria take up excess cytosolic Ca^2+^. This, however, is accomplished at the expense of mitochondrial membrane potential (ΔΨm) [165]. The resulting membrane depolarization can lead to ATP depletion, mitochondrial membrane permeabilization, and finally apoptosis or necrosis [165]. In addition, the mitochondrial Ca^2+^ overload could stimulate the over-production of ROS via activation of membrane permeability transition, cytochrome c release, pyridine nucleotide release, and respiratory inhibition [51, 166].

The balance of mitochondrial fission and fusion can also contribute to TBI-induced brain injury. The imbalance of mitochondrial fusion and fission, particularly excessive mitochondrial fission, has been shown to induce mitochondrial respiratory dysfunction, increased ROS production, and the release of apoptosis-related factors [53–57]. This imbalance of fusion and fission has been evidenced by a significant increase in the length of hippocampal mitochondria 24 h after TBI, followed by significantly decreased length 72 h post-injury [53]. Moreover, the level of mitochondrial fission-related protein Drp1 was significantly upregulated after TBI, indicating an increased mitochondrial fission that would account for the increased mitochondrial length observed [53]. Interestingly, inhibiting mitochondrial fission with the protein inhibitor Mdivi-1 prevented the later decrease in mitochondrial length and reduced the loss of newly formed neurons in the hippocampus [53].

Lastly, it has been reported that mitophagy in TBI is impaired. Damaged mitochondria are accumulated inside the neuronal cells and produce high levels of ROS that cause damage to other mitochondria and eventually induce neuronal death [53].

### Mitochondrial dysfunction in stroke

Ischemic stroke is the most common type of stroke, occurring in more than 87% of cases [167]. It is usually caused by a blood clot-induced obstruction of one or more cerebral arteries in the brain [168, 169]. This obstruction prevents oxygen, glucose, and other nutrients required for cellular homeostasis to reach their destination within the ischemic brain. As a result, energy homeostasis and ATP synthesis are disturbed [168]. In fact, the insufficient supply of oxygen and glucose causes mitochondrial dysfunction within minutes after ischemia, leading to ATP depletion and ROS overproduction [168]. ATP depletion has been shown to induce a cascade of adverse cellular events, including failure of membrane ion pumps, cellular potassium efflux, sodium influx, and membrane depolarization [59–61]. One study has reported that the impairment of ATP-dependent Ca^2+^ channels can induce an overload of Ca^2+^ in the mitochondrial matrix, which further exacerbates ROS production and mitochondrial dysfunction [62, 65].

Excessive ROS production and increased membrane permeability induced by mitochondrial damage can lead to the initiation of the intrinsic apoptotic pathway [66, 170]. This apoptotic process is dependent upon the release of cytochrome c from mitochondria into the cytosol [66]. Once released, cytochrome c, procaspase-9, apoptotic protease activating factor 1 (APAF-1), and dATP form a complex called an apoptosome in the cytosol [66]. The formation of the apoptosome induces the activation of caspase-9, which is followed by the activation of caspase-3 and other executioner caspases that are responsible for accomplishing the apoptotic process [66].

In addition to ROS generation and apoptosis initiation, mitochondrial dynamics has also been implicated in ischemic stroke pathology [168]. The imbalance of mitochondrial fission and fusion has been well documented as an early event required for ischemia-induced neuronal death [70–72]. Mitochondrial fission has been found as early as 3 h after reperfusion in the middle cerebral artery occlusion (MCAO) mouse model for ischemic stroke [73]. As further support, the level of mitochondrial fission proteins Drp1 and Opa1 were found to be upregulated after MCAO [73]. Interestingly, it has been demonstrated that neurons resistant to ischemia can shift mitochondrial dynamics more towards fusion (i.e. less fission), suggesting that the balance of mitochondrial dynamics plays a critical role in neuronal response to ischemic injury [74].

On the other hand, the role of mitophagy in ischemic stroke remains controversial. Accumulating data has indicated that mitopahgy can be protective or destructive after stroke [75, 171–173]. Most studies support that mitophagy activation is a promising therapeutic target for ischemic stroke since mitophagy is able to remove impaired mitochondria and inhibit cell death signaling cascades [76, 77]. However, there are studies that have found that the inhibition of mitophagy confers a neuroprotective effect after MCAO and that excessive mitophagy can cause neuronal death after stroke [75].

Hemorrhagic stroke is the second most common type of stroke. Hemorrhagic stroke occurs when a weakened blood vessel bursts and bleeds into the surrounding brain. Reduced cerebral blood flow has been found in patients with intracerebral hemorrhage as well as experimental models [174–176]. This reduced blood flow has been closely associated with abnormal mitochondrial respiration and decreased basal mitochondrial ATP production [177]. It has also been reported that NMDA receptor is activated after intracerebral hemorrhage, thereby allowing a large Ca^2+^ influx and inducing an excessive production of superoxide through NADPH oxidase and the mitochondrial ETC [63, 64].

### Mitochondrial dysfunction in depression

Increasing evidence implicates mitochondrial dysfunction in the pathogenesis of depression [21]. It is well-known that neurons mainly obtain energy from mitochondrial oxidative phosphorylation and that when oxidative phosphorylation is reduced, the production of ATP cannot meet the energy demand of the cell [178]. Chronic mild stress has been shown to inhibit oxidative phosphorylation, reduce mitochondrial membrane potential, and induce damage to the mitochondrial ultrastructure in the hippocampus, cortex, and hypothalamus of mouse models, thereby indicating a close relationship between mitochondrial dysfunction and depression [78, 179]. Another study found that the utilization of glucose in the prefrontal cortex, cingulate gyrus, and caudate nucleus was decreased in patients with depression, suggesting a decreased production of ATP [80, 180, 181]. In line with this finding, other reports have demonstrated that production of mitochondrial ATP and expression of mitochondrial enzymes are decreased in patients with depression compared with healthy subjects [79, 80]. These results were also demonstrated in muscle tissue from patients with depression [182]. Furthermore, several lines of evidence indicate depression pathology has a close relationship with the inhibition of complexes in the mitochondrial respiratory chain and the activity of Na+, K + -ATPase [81–85].

In addition, there are numerous studies suggesting an intriguing link between mtDNA and depression. In a previous study, 68% of depression patients have mtDNA mutations, compared to 35% of control subjects [80]. Similarly, mtDNA copy number variants were found to be significantly lower in leukocytes from depressive patients than in normal control subjects, while mtDNA oxidative damage was significantly higher [183]. Intriguingly, oxidized mtDNA was found to be able to activate pro-inflammatory cytokines and incite inflammation, which is known to play a critical role in depression [86, 184]. Furthermore, 16 mitochondrial genes (TIMM8B, SLC25A23, SFN, SLC25A30, UCP2, etc.) known to control the production of neuronal ATP and oxidative stress were found to be differentially expressed between depressive patients and healthy subjects [185].

A critical role of oxidative stress in depression has been demonstrated in several studies [186–188]. Since the mitochondrial ETC is the primary source of ROS-induced oxidative stress, ETC dysfunction has been hypothesized to be involved in the development of depression [78]. This oxidative stress is produced when complexes in the ETC incompletely reduce oxygen through the donation of electrons and subsequently generate oxygen radicals including peroxides and superoxide [88]. Excessive radicals are able to damage lipids and proteins, oxidize mtDNA, and incite DNA breaks [88, 89]. This process may play a key role in the pathogenesis of depression.

### Mitochondrial dysfunction in Parkinson’s disease

PD has been recognized as the second most common neurodegenerative disorder and is characterized by a progressive loss of dopaminergic (DA) neurons and the presence of Lewy bodies [189]. Increasing evidence supports mitochondrial dysfunction as one of the main contributors to PD pathogenesis [90]. A study on single neurons from idiopathic PD patients found that the abundances of Complex I and II were typically reduced [91]. Inhibition of Complex I has been shown to induce dopaminergic neurodegeneration in humans, flies, and rodents, indicating a critical role for mitochondrial dysfunction in PD [90, 190, 191]. In line with this, oxidative damage has been found in postmortem brain samples from PD patients [96].

Furthermore, certain mitochondria-related gene mutations capable of inducing mitochondrial dysfunction have been shown to cause familial forms of PD [92]. Mutations in numerous genes, including *SNCA*, *LRRK2*, *Parkin, PINK1,* and *ATP13A2,* have been recognized as monogenic causes of familial PD [93]. These mutations have been directly associated with mitochondrial dysfunction [93]. Additionally, mtDNA in single neurons from idiopathic PD patients presented an increased number of multiple deletions on the background of a common deletion [93]. Consistently, an accumulation of mtDNA mutations and reduced mtDNA copy numbers were found in the substantia nigra from sporadic PD patients [94, 95]. However, the increased mtDNA copy number seen with age in controls was not found in PD patients [93].

In addition to genetic damage and mutations, the malfunction of mitochondrial fission and fusion can cause the death of dopaminergic neuronal cells in PD [98]. A previous study reported that PD-related genes (i.e. *PINK1* and *Parkin*) play pivotal roles in regulating the balance of mitochondrial fission and fusion [99]. In a recent study using a neurotoxin model of sporadic PD, increased nitric oxide levels induced Parkin nitrosylation, resulting in the reduced ability of Parkin to suppress Drp1 and thereby causing mitochondrial hyper-fragmentation [100].

Mitochondrial calcium overload was also found in PD. In dopaminergic neurons, excessive Ca^2+^ released from the endoplasmic reticulum impacted mitochondrial Ca^2+^ homeostasis, resulting in mitochondrial dysfunction and an apoptotic cascade [101]. Moreover, inhibition of mitochondrial Ca^2+^ overload was found to be able to render a neuroprotective effect in zebrafish models of PD [102].

Similar to other brain diseases, emerging evidence suggests that inappropriate trafficking of damaged mitochondria and compromised mitophagy contribute to mitochondrial dysfunction and PD pathogenesis [103, 104].

### Methylene blue and Photobiomodulation as therapeutic approaches

A large body of evidence suggests a role for mitochondrial dysfunction in the pathogenesis of several brain diseases. As a result, much attention has been directed towards developing therapies for these diseases by targeting mitochondria and cellular respiration. Methylene blue and photobiomodulation are two such therapies.

Methylene blue (3,7-bis (dimethylamino)-phenothiazin-5-ium chloride, MB) is an FDA-approved medication which has been used as an effective agent in malaria treatment, methemoglobinemia, and cyanide poisoning [192, 193]. Recently, the potential role of MB in the treatment of neurodegenerative disorders, ischemic brain injury, and TBI has captured researchers’ attention [9, 17, 22, 194–197]. Furthermore, its beneficial effects on psychosis has been reported in preclinical and clinical studies [9, 17, 22, 195–197]. In AD patients and AD animal models, cognitive performances were significantly improved after MB treatment [198, 199]. According to a randomized, double-blinded, placebo-controlled clinical trial, low-dose MB was able to increase functional MRI activity during a short-term memory task and also improved memory retrieval [200]. In addition, MB has been tested in a human clinical trial in which patients with mild to moderate AD showed both cognitive and cerebral blood flow improvements after MB treatment [201]. The potential therapeutic role of MB for neurological disorders may result from a transformation between the reduced and the oxidized forms of MB [193]. During this process, MB can easily cross the blood-brain barrier and donate electrons from its reduced form to the mitochondrial electron transport chain (ETC), thereby increasing oxygen consumption and ATP formation [22, 202].

Intravenous administration of MB allows for higher available concentrations of the drug than oral administration, and therefore is the optimal means of delivery [201]. Following administration, MB can accumulate in various tissues at significant concentrations, with brain tissue concentration of MB being as much as 10 times higher than serum levels 1 hour post-injection [201]. The substantial accumulation in the brain allows MB to easily cross the BBB and preferentially enter neuronal mitochondria, although the mechanism for mitochondrial penetration is unclear [202]. Because MB readily crosses the BBB, has a strong affinity for mitochondria, and acts as a powerful antioxidant, the FDA approved MB to be routinely prescribed as an antidote for the treatment of poison-induced methemoglobinemia [203–205].

Photobiomodulation (PBM), originally known as “low-level laser therapy (LLLT)”, was first described almost 50 years ago and refers to the application of red-beam (400–720 nm) or near-infrared (700–1000 nm) laser on biological tissues [206]. PBM therapy can modulate various biological processes and confer a protective effect against tissue damage and cell injury [8, 207, 208]. For example, studies found that LLI could promote the regeneration of gastrocnemius muscle after cold injury and enhance neovascularization after injury, indicating a potential role of PBM on regeneration and angiogenesis after muscle injury [209, 210]. In addition, the beneficial effects of PBM have been experimentally demonstrated on recurrent aphthous stomatitis, skin burn injuries, diabetes mellitus, sports injuries, and osteoarthritis [211–216]. Most significantly, PBM is able to confer its photobiological effect at the cellular level without thermal and toxic effects [217]. Interestingly, mitochondria are considered to be the target of PBM, wherein low-level laser donates photons to Complex IV, thereby increasing the activity of Complex IV and subsequent oxygen consumption [202, 218].

### Methylene blue, Photobiomodulation and AD

In AD, it has been found that mitochondrial malfunction often occurs before obvious plaque deposition and memory defects [219]. Previous studies have demonstrated an association between mitochondrial dysfunction and Aβ accumulation [31, 220, 221]. Accumulated Aβ has been shown to be able to bind to the mitochondrial enzyme amyloid-binding alcohol dehydrogenase (ABAD), an enzyme responsible for the conversion of estradiol to estrone, a process which is important for mitochondrial protection [222–224]. This binding of Aβ and ABAD has been shown to induce ABAD structural changes and result in the formation of an Aβ-ABAD complex, leading to changes in mitochondrial membrane permeability and decreased respiratory enzyme activity [225]. However, interestingly, MB has been shown to decrease ABAD overexpression, decrease Aβ levels, and therefore decrease Aβ-ABAD binding, thereby preserving ABAD functions, attenuating mitochondrial dysfunction, and conferring a positive effect on Aβ accumulation [226].

An additional beneficial effect of MB on AD is the clearance of Aβ through increased activity of chymotrypsin and trypsin-like proteasome [199]. Previous studies treating 3xTg-AD and APP/PS1 mouse models with MB demonstrated a decreased deposition of Aβ and improvement of learning and memory through chymotrypsin and trypsin-like proteasome activation and β-secretase activity attenuation [198, 199, 227]. Similar results were found on the transgenic PSAPP mice wherein MB was reported to be able to attenuate the activity and expression of β-secretase [198]. In this way, MB has been shown to inhibit the formation of neurotoxic oligomeric Aβ and improve behavioral results [19, 20].

NFT aggregated by hyperphosphorylated tau protein (p-tau) is another pathological hallmark of AD [228]. MB has been reported to inhibit this aggregation by attenuating tau-tau interactions [229]. Furthermore, MB is also able to prevent the formation of tau filament by acting on the microtubule binding domain [230]. In a previous in vitro study, the N-unsubstituted phenothiazine ring of MB was found to be necessary for the inhibition of filament formation [230]. In a clinical study, patients with mild to moderate AD took MB orally at 60 mg three times daily for 24 months [229]. Results showed that patients with MB showed an 81% reduction in the rate of cognitive decline after 50 weeks compared with controls [229]. The authors suggested that the beneficial effect of MB on AD patients was linked to the prevention of tau aggregation [229].

In addition, MB has been suggested to directly interact with the mitochondrial ETC as a catalytic redox cycler carrying electrons from NADH to Complex IV [17, 22]. During the conversion between MB and its reduced counterpart Leuco MB, excessive ROS is reduced by bypassing the pathological blockage of Complex I and III [17, 22]. MB is also known to be able to enhance the activity of Complex IV as well as upregulate heme synthesis, and thereby promote mitochondrial function in the AD brain [231–233].

The neuroprotective effect of PBM on AD has also been well established over the past few decades. In an amyloid-β protein precursor (AβPP) transgenic mouse model, the amount of Aβ plaques and amyloid was significantly reduced and was accompanied by improved behavioral results [234]. In another transgenic mouse model (K369I tau transgenic model), the levels of hyperphosphorylated tau and neurofibrillary tangles were significantly decreased as was the expression of oxidative stress markers [235]. Recently, a new perspective on the mechanism of PBM in AD treatment was proposed, in which mesenchymal stem cells stimulated by PBM were shown to maturate towards a monocyte lineage, thereby increasing the ability of Aβ phagocytosis in vitro [236]. Furthermore, PBM has been shown to improve spatial learning and memory ability by significantly reducing Aβ burden in the brain [236]. An in vitro study reported that PBM was able to exert neuroprotection against Aβ toxicity and inhibit dendritic spine loss in neurons by activating the ERK/CREB pathway and upregulating the expression of BDNF [237].

Similarly to MB, the effect of PBM on AD occurs mechanistically through the improvement of mitochondrial function [40, 238]. In a previous study, we demonstrated the beneficial effects of PBM on the streptozotocin (STZ) induced AD model [8]. These beneficial effects occurred via several mitochondria-related mechanisms. First, we showed that PBM can inhibit the expression of mitochondrial fission-related proteins and improve the expression of fusion-related proteins, which directly improves mitochondrial dynamics [8]. Secondly, the restoration of mitochondrial dynamics facilitated mitochondrial homeostasis by decreasing the Bax/Bcl-2 ratio elevated by STZ injection [8]. Furthermore, our study demonstrated that PBM was able to suppress the oxidative damage and inflammation seen in AD [8].

### Methylene blue, Photobiomodulation and TBI

Since mitochondrial dysfunction is well studied in TBI, the ability of MB to treat mitochondrial dysfunction as a means of neuroprotection has been the subject of investigation. In addition to the beneficial effects seen in other neurological diseases, MB has been found to be able to reduce TBI-induced edema, attenuate lesion volume, and improve behavioral scores [163]. In previous studies, MB has been reported to be able to cross the blood-brain barrier and accumulate in the brain with 10–20 times higher concentration than that in the circulation after intravenous injection [163]. No side effects have been reported in animals given a low dose of MB (0.5–5 mg/kg) [239, 240]. However, it must be noted that adverse effects can occur with high doses of MB (> 10 mg/kg) due to the interference with the ETC [239].

In a study using a low dose of MB (1.5 mg/kg) on the first day of TBI, edema was shown to significantly decrease in the injured hemisphere [241, 242]. This sort of edema reduction is critical in reducing TBI-associated mortality. The effect of MB on the number of surviving neurons has also been determined by a previous study. At 24 and 72 h post-TBI, the number of surviving neurons after MB treatment was significantly increased compared with the group treated with vehicle injection. In addition, increased Belin 1 expression and an increased LC3-II to IL3-I ratio were found in MB groups, indicating an increased induction of autophagy [241].

Recently, a study using in vitro cell (oxygen glucose deprivation/reoxygenation injury, OGD) and TBI animal models determined whether MB treatment was able to confer beneficial effect on neuronal mitochondrial dysfunction. In the OGD model, they demonstrated MB treatment was able to inhibit excessive neuronal ROS production, maintain mitochondrial membrane potential, and induce increased ATP generation, indicating that MB is able to attenuate OGD induced-mitochondrial dysfunction [243]. This was further confirmed by blood brain barrier protection and decreased cytochrome c release and neuronal apoptosis in the TBI animal model after MB treatment [243].

In addition to MB, PBM has been suggested as a possible therapeutic strategy for TBI since PBM has been reported to increase mitochondrial function, improve blood flow, reduce swelling, decrease oxidative stress, inhibit inflammation, and attenuate apoptosis [244]. Exposing an injured mouse head (TBI) to PBM with a near-infrared laser (808 nm) has been shown to improve neurological performance and decrease lesion size [245]. Interestingly, laser wavelength seems to play a role in PBM efficacy since PBM with 665 nm and 810 nm wavelength lasers caused significant neurological improvement while PBM with 730 nm and 980 nm wavelength lasers did not [246]. This phenomenon may be explained by the target of the laser light. Complex IV is proposed to absorb laser light between 665 nm and 810 nm, suggesting that it is the target of PBM treatment [246]. An elevation of ATP production following PBM treatment in mice with mild head TBI supports the proposed role of the mitochondrial ETC as the cellular target for PBM [247].

The efficacy of PBM on TBI has also been shown in TBI patients. Learning memory, mathematical skills, and neuropsychological test results were significantly improved after 9 months of PBM treatment [248]. Moreover, a patient who had suffered severe TBI could spontaneously move his arm and hand after 5 days of PBM treatment [249].

### Methylene blue, Photobiomodulation and stroke

Another neurological disorder for which MB and PBM has been proposed is stroke [16, 250]. In a focal cerebral ischemia rat model, behavioral results were significantly improved after chronic oral MB treatment [16]. Further studies demonstrated its beneficial role in decreasing total lesion volume, cerebral edema, and gray and white-matter damage, all of which contributed to the improvement of behavioral results [16, 18]. Lin et al., demonstrated that MB treatment was able to improve the activity of mitochondrial Complexes I, II, and III and significantly increase both oxygen consumption and glucose uptake in HT22 cells, a well-known neuronal cell line [251]. Their in vivo experimental results indicated that cerebral global glucose uptake and blood flow were significantly increased in animals treated with MB [251]. Furthermore, an in vitro study found that oxygen consumption rate increased after MB treatment even under the inhibitory effect of Complex I, III, and V inhibitors [252]. In addition, neuronal ATP production has been shown to improve with MB treatment [252]. In a MCAO rat model, the decreased activity of Complex I, III, and IV after ischemia/reperfusion injury contributed to decreased mitochondrial function, which was restored with a low-dose of MB [252].

One of our previous studies found that MB was able to attenuate stroke-induced behavioral defects and improve neurogenesis in the peri-infarct area by increasing mitochondrial function [9]. The underlying mechanism for this recovery was the improvement of the mitochondria-dependent microenvironment around newborn neurons [9].

Furthermore, in an acute cerebral ischemic injury model, improvement of neurological function was determined to be related to the augmentation of mitophagy wherein MB was able to attenuate the ischemia-induced disintegration of the mitochondrial structure [253]. However, the effects of mitophagy in stroke remains controversial. The neuroprotective role of MB in cerebral ischemia has also been suggested to be caused by increased mitophagy and the maintenance of mitochondrial membrane potential [253].

Similarly to MB, PBM therapy has been proposed as a promising therapeutic approach for stroke [254]. Previous studies on animals have demonstrated that PBM treatment using 660-808 nm low-level laser improved neurological rating scores without thermal effects [4, 255, 256]. Similarly, the safety and efficacy of using infrared laser therapy within 24 h of stroke onset has been validated in a human patient study [257]. In line with these studies, a large body of research on LED-PBM using in vitro cell assays has been published [258, 259]. In both human epithelial cells (HEP-2) and mouse subcutaneous connective tissue cells (L-929), PBM treatment increased metabolism and proliferation [258]. The association between ischemic stroke and dementia has been well studied in previous studies [260, 261]. Interestingly, PBM also displays protective effects in dementia patients after ischemic stroke by stimulating cerebral neurogenesis [262]. In a study using a photothrombotic (PT) model of ischemic stroke, PBM was observed to improve behavioral results by enhancing mitochondrial function and neurogenesis [4].

Similarly to MB, neuronal mitochondria are regarded as the target for PBM. PBM initiated 6 h after GCI was shown to protect against CA1 neuronal cell death through the preservation of healthy mitochondrial dynamics and the suppression of mitochondrial fragmentation [263]. During this process, the preservation of mitochondrial integrity by PBM attenuated mitochondrial oxidative damage and excess mitophagy and suppressed mitochondria-dependent apoptosis [263]. In a study using a neonatal animal model, hypoxia ischemia (HI)-induced brain lesions in the cortex and hippocampus were significantly decreased after PBM treatment [254]. Further mechanistic investigations indicated that PBM could attenuate HI-induced mitochondrial fragmentation and significantly restore mitochondrial dynamics [254]. In addition, PBM inhibited HI-induced mitochondrial membrane collapse, improved ATP production, and decreased protein carbonylation, DNA oxidative damage, and lipid peroxidation [254].

### Methylene blue, Photobiomodulation and depression

In an early study in 1983, MB was recognized as a potential method for manic depression [197]. In their study, oral MB administration significantly improved the symptoms of 14 of 19 manic depressive patients for whom standard therapies failed [197]. In line with this, MB was also shown to be an effective addition in the long-term treatment of depression in a double-blind clinical trial [264]. Moreover, MB can work effectively even on severe depression. The symptoms of patients included in a controlled trial with severe depressive illness were significantly improved after receiving 15 mg/day MB treatment [265]. Previous studies have also confirmed the efficacy of MB for the treatment of bipolar disorder [266]. Furthermore, depressive-like behavior in both human patients and animal models can been observed after TBI [267, 268]. However, MB has been shown to inhibit this depressive-like behavior [269].

These improvements may be explained mechanistically by the role of nitric oxide (NO), an unconventional gaseous neurotransmitter, in mood disorders and by the selective inhibition of nitric oxide synthase (NOS) [264, 265, 270, 271]. Not only does MB inhibit NOS in the brain, but it also affects other heme-containing enzymes including various cytochromes [272, 273].

PBM has also been suggested to have a potential antidepressant role. In a study of reserpine-induced depression, the results of the forced swimming test, which tests mobility, improved after PBM treatment [274]. In addition, after several weeks of laser acupuncture intervention, symptoms improved significantly in depressive patients [275, 276]. Furthermore, depressive symptoms evidenced improvement after transcranial (t-PBM) and intranasal (i-PBM) treatment in a case report on a 76-year-old female with major depressive disorder [277]. The beneficial effects of PBM on depression have also been demonstrated in a space restriction-induced depression animal model and in Abelson helper integrationsite-1 (Ahi1) KO mice [208]. In both of these two mouse models of depression, depressive behaviors were effectively improved by PBM [208]. Further mechanistic investigation suggested that ATP production and the activity and expression of mitochondrial Complex IV were significantly improved after PBM treatment [208]. Therefore, PBM may attenuate behavioral deficits in depression by improving energy metabolism.

### Methylene blue, Photobiomodulation and Parkinson’s disease

As mentioned earlier, PD, the most common movement disorder, is characterized by mitochondrial dysfunction although the underlying mechanism contributing to the dopaminergic neuronal damage remains unclear [90]. However, mitochondrial dysfunction-induced oxidative stress has been closely linked to the loss of neurons in PD [278, 279]. In a 6-OHDA-induced PD model, 6-OHDA administration was performed to induce dopamine depletion and cause disruption of mitochondrial function and excessive production of ROS [280–283]. Because of the antioxidant properties of MB, a research group anticipated that MB administration could confer neuroprotective effects on substantia nigra pars compacta (SNc) dopamine cells [284]. Although it was not accompanied by improved behavioral results, a low dose of orally administered MB mitigated 6-OHDA-induced SNc dopamine cell loss [284].

In rotenone model of PD, similar results were found when MB was able to attenuate rotenone-induced inhibition of mitochondrial Complexes I, II, and III, reduce free radical production, and improve behavioral results [252, 285]. A study using an MPTP (1-methyl-4-phenyl-1, 2, 3, 6-tetrahydropyridine)-induced PD animal model to evaluate the effect of MB on dopamine cells found that MB could cause the upregulation of brain-derived neurotrophic factor (BDNF) and induce the activation of its downstream signaling pathways, indicating BDNF may be one of the contributing factors for MB-mediated neuroprotection [286].

6-OHDA and MPTP-induced PD animal models have also been widely used in the study of PBM treatment of PD. In these models, PBM treatment was able to fully rescue lesions with 15% cell loss [287, 288]. However, lesions with up to 50% cell loss were unrecoverable [289]. These results were confirmed in a lipopolysaccharide (LPS)-induced dopaminergic cell loss rat model [290]. PBM could fully rescue rats from 15% LPS-induced cell loss by inhibiting inflammatory amoeboid microglia, however, there was no significant neuroprotection on groups with 50% cell loss [290].

PBM has also been used in a randomized clinical trial in which the heads of PD patients were exposed to red light-emitting diodes (LED) for a 9-week time course [291]. After 9-weeks treatment, there was no significant difference in the sham group [291]. However, PBM groups presented significant gait improvement [291]. Another clinical report showed an improvement in speech, cognition, gait, and freezing episodes in PD patients administered 2 weeks of PBM therapy [292]. Given evidence from studies on PBM treatment for other neurodegenerative disease, it is plausible that the mechanism underlying the beneficial effect of PBM on PD could be due to improved mitochondrial function and reduced oxidative stress [293].

### Primary mechanisms underlying mitochondrial protection through MB and PBM

**Fig. 2:**
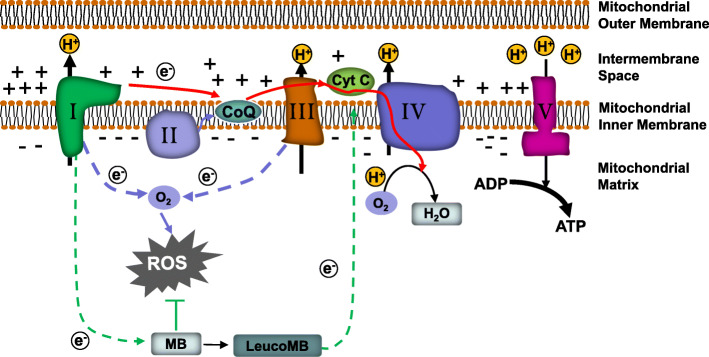
Primary Mechanisms Underlying Mitochondria Protection through MB. MB reroutes the pathway of electron transfer by working as an alternative electron transporter. By bypassing the ETC between Complex I and Complex III, MB efficiently attenuates electron leakage and subsequent ROS generation

As reported, both MB and PBM are known to enhance energy production and decrease oxidative stress [294–296]. However, the underlying mechanism for these effects differs between the two treatments. As shown in Fig. 2, MB works as a catalytic redox cycler which is reduced in the mitochondrial matrix by electrons donated from NADH [297, 298]. This results in the formation of MBH_2_, also called Leuco MB. BH_2_ is able to bypass the compromised ETC between Complex I and Complex III and readily recycle between its reduced form and oxidized form due to its low redox potential [297, 298].

As mentioned previously, mitochondria are the major site for ROS generation. Under pathological conditions, Complex I and Complex III act as the main sites for electron leakage in mitochondria [22]. The electrons leaking from Complex I and Complex III are transferred to O_2_, reducing it to O2• − [299]. The excessive ROS will primarily damage Complex I and Complex IV, which will induce additional mitochondrial dysfunction [299, 300]. Moreover, high levels of ROS and oxidized mitochondrial DNA can induce the activation of the NLRP3 inflammasome, which triggers an innate immune response through the release of pro-inflammatory cytokines [142, 301–303]. However, the oxidation/reduction of MB can act as an alternative mitochondrial electron transfer pathway. By bypassing Complex I and Complex III, MB efficiently attenuates electron leakage and subsequent ROS generation [22]. Due to the reduction of ROS, MB can also play a role in the inhibition of inflammation. Although several mechanisms underlying the neuroprotective effects of MB have been reported, including caspase-6 activity inhibition [304], restoration of PMCA pump function [305], PI3K/Akt/GSK3β pathway activation [306], reduction of pro-inflammatory cytokines [286], attenuation of synaptic toxicity and MARK4/PAR1-mediated tau phosphorylation [286], Nrf2/ARE signaling pathway activation and MEF2D-associated survival pathway induction [307], the possibility that mitochondria are involved in those processes cannot be excluded [304].

The mechanism for the beneficial effects of PBM on mitochondrial function, however, is quite different and depends on chromophores located within the cells. According to the first law of photobiology, PBM must be absorbed by a chromophore within the tissue of interest in order to confer biological effects; mitochondria play a key role in this light absorption [308].

Complex IV (cytochrome c oxidase, CCO) in the ETC contains 13 separate protein subunits with two heme moieties (heme α and hem α_3_) and two redox-active copper centers (Cu_A_ and Cu_B_) along with one zinc and one magnesium center, which are all believed to be absorbing chromophores for visible light [295, 309]. In the process of ATP generation, Complex IV transfers four protons along with the electrons from reduced cytochrome c to oxygen to form two H_2_O molecules [22, 295]. This process contributes to the formation of the proton gradient which drives the activity of ATP synthase [22, 295].

**Fig. 3:**
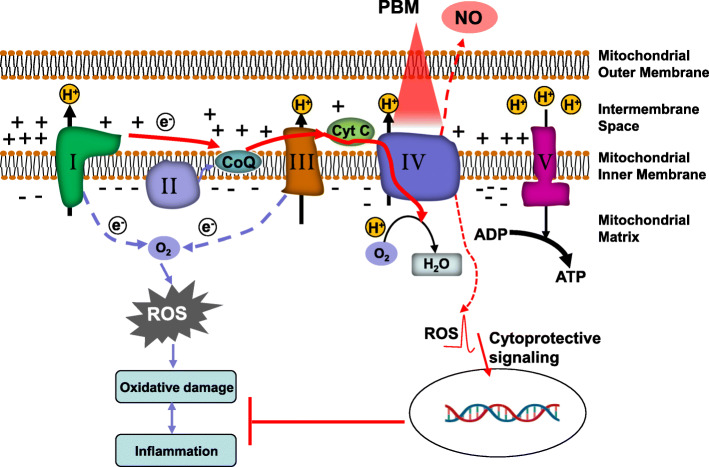
Primary Mechanisms Underlying Mitochondria Protection through PBM. PBM treatment causes NO to dissociate from Complex IV (cytochrome c oxidase, CCO), causing the complex’s activity to increase. This allows the flux of electrons, the pumping of protons, and the synthesis of ATP to increase, thereby boosting cellular energy levels. In addition, when PBM stimulates Complex IV activity in normal cells, mitochondrial membrane potential is increased above normal baseline levels, resulting in a brief and rather modest increase in ROS production. The short burst of ROS is able to activate cytoprotective signaling, which attenuate ROS induced oxidative damage and neuroinflammation

According to previous studies, PBM is considered to enhance the activity of Complex IV and subsequently improve the generation of ATP (Fig. 3) [295]. This enhanced activity has been suggested to be due to a PBM-mediated photodissociation of NO from Complex IV [310]. NO is a molecule inhibiting Complex IV by non-covalently binding to heme α3 and CuB [310, 311]. When NO is photodissociated from Complex IV, the activity of Complex IV is enhanced, improving energy production [310]. Under normal conditions, ROS is generated at low levels by normal mitochondrial metabolism [310]. When PBM stimulates Complex IV activity in normal cells, mitochondrial membrane potential is increased above normal baseline levels, resulting in a brief and rather modest increase in ROS production [312]. The short burst of ROS is able to activate NF-kB in the cytoplasm [312]. The released NF-kB will be transported from the cytoplasm to the nucleus, where it will induce the expression of more than 150 genes, including genes related to mitochondrial dynamics, inflammation, and antioxidant activity [217].

In pathological states, the increased mitochondrial membrane potential mediated by PBM is able to lower ROS production [217, 295]. As result, pro-inflammatory NF-kB activity is lowered. Evidence has demonstrated that PBM is able to attenuate the levels of pro-inflammatory cytokines produced from activated inflammatory cells [313–315]. This anti-inflammatory effect of PBM may due to its ability to mediate the transformation between the M1 “pro-inflammatory” phenotype and the M2 “anti-inflammatory” phenotype of microglia although the exact mechanism is unclear [4, 206].

## Conclusion

**Fig. 4:**
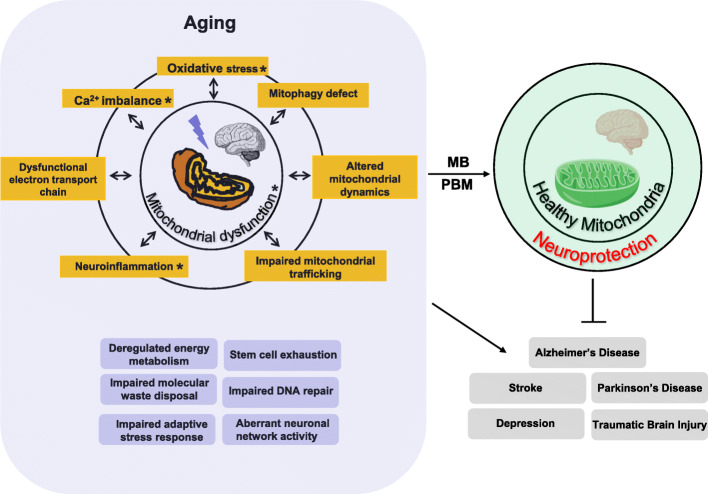
Mitochondrial related changes associated with mitochondrial dysfunction. Mitochondrial dysfunction, oxidative damage, dysregulated neuronal Ca^2+^_,_ and neuroinflammation are hallmarks of brain aging (the other six hallmarks are deregulated energy metabolism, stem cell exhaustion, impaired molecular waste disposal, impaired DNA repair, impaired adaptive stress response, and aberrant neuronal network activity) . Increasing evidence suggested the hallmarks of brain aging affect several brain disease etiologies [316, 317]. MB and PBM can attenuate the pathological symptoms of several brain diseases and lead to neuroprotection by reducing various aspects of mitochondrial dysfunction

MB and PBM are two promising therapeutic approaches for brain disorders. Both MB and PBM target mitochondria, although their underlying mechanisms differ (Table 2). The studies discussed offer compelling evidence that suggests mitochondrial dysfunction could be a potential target for neuroprotection for several brain disorders. The mitochondrial dysfunction-related processes that could be targeted and improved upon include impaired ETC function, excessive ROS production, oxidative damage, Ca^2+^ overload, aberrant mitophagy, altered mitochondrial dynamics, impaired mitochondrial trafficking, and subsequent neuroinflammation (Fig. 4), of which most are included in the 10 hallmarks of brain aging reported recently [316, 317]. Since MB and PBM both target mitochondria through distinct mechanisms, a therapy combining their use may be able to ameliorate the symptoms of brain disease beyond the ability of either individual therapy. Such a combined therapy warrants serious future investigation in animal studies and clinical trials. If proven effective, the combined use of MB and PBM may provide a promising new avenue for the treatment of multiple brain diseases.

**Table 2:** Summary of effects of MB or PBM on neurodegenerative disorders and brain injury

Disease	Effect of a treatment with MB	Effect of a treatment with PBM
**AD**	• Increases functional MRI activity and improves memory retrieval [200]	• Reduces hyperphosphorylated tau, neurofibrillary tangles, and oxidative stress [234, 235]
	• Decreases Aβ levels and Aβ-ABAD binding [226]	• Increases the ability of Aβ phagocytosis [236]
	• Attenuates the activity and expression of β-secretase, inhibits the formation of neurotoxic oligomeric Aβ, and improves behavioral results [19, 20, 198]	• Improves spatial learning and memory by significantly reducing Aβ burden [236]
		• Exerts neuroprotection by activating the
	• Inhibits p-tau aggregation and tau-tau interactions [229, 230]	ERK/CREB pathway and upregulating the expression of BDNF [237]
	• Reduces excessive ROS production [17, 22]	
	• Upregulates Complex IV activates, heme synthesis and mitochondrial function [226, 231–233]	• Restores mitochondrial dynamics [8]
**TBI**	• Decreases edema and lesion volume and improves behavioral scores [163]	• Neurological improvement [246]
	• Increases autophagy [242].	• Increases mitochondrial function, improves blood flow, and reduces swelling [244, 247]
	• Inhibits excessive ROS production and attenuates mitochondrial dysfunction, cytochrome c release, and neuronal apoptosis [243]	
		• Decreases oxidative stress, inhibits inflammation, and attenuates apoptosis [244, 247]
**Stroke**	• Improves behavioral results after focal cerebral ischemia [16]	• Improves neurological rating scores [4, 255, 256]
	• Decreases lesion volume, cerebral edema, and gray and white- matter damage [16, 18]	• Stimulates neurogenesis and improves mitochondrial function [4, 262]
	• Increases cerebral global glucose uptake and blood flow [251, 252]	• Preserves mitochondrial integrity [263]
	• Increases mitochondrial function [9, 252]	• Attenuates mitochondrial fragmentation and restores mitochondrial dynamics [254]
	• Preserves mitochondrial structure and function [253]	
	• Increases mitophagy and preserves mitochondrial membrane potential [253]	• Decreases protein carbonylation, DNA oxidative damage, and lipid peroxidation [254]
**Depression**	• Improves the symptoms of patients with severe depression [265]	• Improves depressive symptoms [274–277]
	• Selectively inhibits nitric oxide synthase (NOS) [264, 265, 270, 271]	• Improves ATP production and increases activity and expression of mitochondrial Complex IV [208]
**PD**	• Attenuates dopamine loss and reduces the disruption of mitochondrial function and excessive production of ROS [280–284]	• Reduces cell loss and inhibits inflammatory amoeboid microglia [287–290]
	• Improves Complexes I, II, and III activities, reduces free radical production, and improves behavioral results [252, 285]	• Improves speech, cognition, gait, and freezing episodes in PD patients [291, 292]
	• Upregulates brain-derived neurotrophic factor (BDNF) expression [286]	• Improves mitochondrial function and reduces oxidative stress [293]

## Data Availability

Not applicable.

## Abbreviations

ABAD: Amyloid-binding alcohol dehydrogenase
AD: Alzheimer’s disease
Ahi1: Abelson helper integrationsite-1
ALS: Amyotrophic lateral sclerosis
APE-1: Apoptotic protease activating factor 1
APP: Amyloid precursor protein
ATP: Adenosine triphosphate
Aβ: Amyloid-β
AβPP: Amyloid-β protein precursor
BBB: Blood-brain barrier
BDNF: Brain-derived neurotrophic factor
CAT: Catalase
Cyt c: Cytochrome c
DA: Dopaminergic
DAMPs: Damage-associated molecular patterns
ETC: Electron transport chain
GPx: Glutathione peroxidase
GR: Glutathione reductase
GSH: Glutathione
HEP-2: Human epithelial cells
HI: Hypoxia ischemia
LED: Light-emitting diodes
LPS: Lipopolysaccharide
MB: Methylene blue
MCAO: Middle cerebral artery occlusion
MCU: Mitochondrial calcium uniporter complex
Mfn2: Mitofusin 2
MPTP: 1-methyl-4-phenyl-1,2,3,6-tetrahydropyridine
mtDNA: Mitochondrial DNA
NFT: Neurofibrillary tangles
NO: Nitric oxide
NOS: Nitric oxide synthase
PBM: Photobiomodulation
PD: Parkinson’s disease
PINK1: PTEN-induced kinase 1
PT: Photothrombotic
p-tau: Hyperphosphorylated tau protein
ROS: Reactive oxygen species
SNc: Substantia nigra pars compacta
SOD: Superoxide dismutase
STZ: Streptozotocin
TBI: Traumatic brain injury
TNFα: Tumor necrosis factor alpha
